# Chest radiographs and machine learning – Past, present and future

**DOI:** 10.1111/1754-9485.13274

**Published:** 2021-06-25

**Authors:** Catherine M Jones, Quinlan D Buchlak, Luke Oakden‐Rayner, Michael Milne, Jarrel Seah, Nazanin Esmaili, Ben Hachey

**Affiliations:** ^1^ I‐MED Radiology Network Brisbane Queensland Australia; ^2^ Annalise.ai Sydney New South Wales Australia; ^3^ School of Medicine The University of Notre Dame Australia Sydney New South Wales Australia; ^4^ Harrison.ai Sydney New South Wales Australia; ^5^ Australian Institute for Machine Learning The University of Adelaide Adelaide South Australia Australia; ^6^ Department of Radiology Alfred Health Melbourne Victoria Australia; ^7^ Faculty of Engineering and Information Technology University of Technology Sydney Sydney New South Wales Australia

**Keywords:** chest X‐ray, clinical decision support, deep learning, machine learning, radiomics

## Abstract

Despite its simple acquisition technique, the chest X‐ray remains the most common first‐line imaging tool for chest assessment globally. Recent evidence for image analysis using modern machine learning points to possible improvements in both the efficiency and the accuracy of chest X‐ray interpretation. While promising, these machine learning algorithms have not provided comprehensive assessment of findings in an image and do not account for clinical history or other relevant clinical information. However, the rapid evolution in technology and evidence base for its use suggests that the next generation of comprehensive, well‐tested machine learning algorithms will be a revolution akin to early advances in X‐ray technology. Current use cases, strengths, limitations and applications of chest X‐ray machine learning systems are discussed.

## Introduction

The discovery of the X‐ray by Wilhelm Rontgen in 1895 quickly led to the application of chest radiography and fluoroscopy to diagnose numerous chest diseases, including tuberculosis, pneumonia and pneumothorax.^1^ This diagnostic leap forward quickly established the chest X‐ray (CXR) as an essential component of the diagnostic pathway for chest disease.

For the next half‐century, extensive work identified and validated anatomical and pathological signs on the chest radiograph, leading to the principles of CXR interpretation used today. X‐ray imaging as a diagnostic tool advanced quickly from initial work to describe imaging appearances of different diseases, later validated in large trials, to the physics of X‐ray production and radiation safety. CXR is now extensively used across medical practice, from the acute setting to disease surveillance and screening. It accounts for around 30–40% of all X‐ray investigations conducted and compared to other imaging techniques, it is fast, widely available and inexpensive with a low radiation dose.^2^, ^3^ It is used globally as a first‐line imaging tool for chest assessment.

Despite the ease with which chest radiographs can be obtained, their interpretation can be challenging. The CXR is fundamentally a 2‐dimensional representation of a 3‐dimensional anatomical structure. X‐rays are absorbed by multiple structures as they pass through the thorax, with the overall attenuation of each ray producing the different pixel values in the X‐ray image. The composite attenuation of each X‐ray beam limits the assessment of the image; ribs and mediastinum obscure up to 40% of the lung parenchyma,^4^ and depending on where a pathological lesion is sited, the differences in density between the pathology and adjacent normal structures may be subtle. This may be exacerbated when patient positioning or the degree of inspiration is suboptimal, medical devices or external objects are in the field of view, or in patients with a larger body habitus.

Even with experienced radiologists and technological advancements in chest radiography, the reported error rates for CXR interpretation have remained constant for decades.^5^, ^6^, ^7^ This may be at least partially due to the unchanging principles of CXR interpretation used by radiologists and other clinicians. The effect of non‐image factors such as fatigue, interruptions to reporting, environmental factors (temperature, lighting and ergonomics), information system delays or failures, staffing issues and workload may also contribute to radiologist error.^8^ Error reduction is one of the key driving factors behind the intense interest in machine learning‐driven diagnostic tools to facilitate CXR interpretation.

Biases and non‐image factors affecting radiologist performance, including satisfaction of report, satisfaction of search,^9^ fatigue, interruptions and the work environment, do not influence machine learning models. A machine learning model may assess the image for all target findings, irrespective of the clinical presentation, underlying disease processes, complexity of the anatomy or study acquisition parameters. Similarly, while radiologists vary in experience and capability (residents, consultants and subspecialists), a model can perform with high accuracy consistently.

We summarise current standards in CXR machine learning, outline factors that lead to successful delivery of CXR machine learning and look to the future of technology and radiology practice.

## Machine learning applications in CXR interpretation

Over the last decade, advances in machine learning technology have led to the development of many new algorithms, including those intended to assist clinicians in interpreting CXR. Digitisation of radiology has allowed for the curation of large, data‐rich image collections that are well suited for training deep convolutional neural networks (CNNs). CNNs are commonly used for image interpretation and are based loosely on the functioning of complex neural networks in the human brain.^10^, ^11^ CNNs are able to recognise salient clinical features in images once trained on a large data set. While the requirement for large volumes of data has previously been a barrier to effective training, recent years have seen compelling applications developed using well‐curated, high‐volume CXR data sets.^12^

Convolutional neural networks have now been applied to CXR analysis to successfully detect a wide range of clinical findings. Assessment of diagnostic performance is based on the calculation of many metrics, the most common of which is the area under the receiver operating characteristic curve (AUC). This is a summary statistic indicating diagnostic accuracy independent of disease prevalence in the testing data set. Sensitivity and specificity are well‐recognised metrics in clinical practice and are also often reported. The Matthews correlation coefficient (MCC) has been highlighted as a preferred metric for binary classification.^13^ Model performance may be compared to that of clinicians and clinician performance with and without model assistance can be assessed.

## Narrow vs comprehensive machine learning models

One useful way to characterise machine learning models is by distinguishing between those that are ‘narrow’ and those that are ‘comprehensive’. Narrow models are trained to complete a single or small number of clinical diagnostic tasks for a given image modality. Whereas comprehensive models are trained to assess an image modality in its entirety for many clinical findings, completing most or all of the tasks that a human expert would be expected to perform in clinical practice.

Many narrow machine learning models have been developed to detect a single finding. The rationale for these has been based on clinical need for particularly salient findings. For example, lung cancer is the most common cancer worldwide and the most common cause of cancer death, with a poor prognosis overall.^14^ While computed tomography has greater sensitivity for lung cancer detection in screening programs, the widespread use of CXR across medicine means that it often provides the first opportunity for early diagnosis. However, ninety per cent of missed lung cancers are due to CXR diagnostic errors.^15^ Recent evidence suggests that machine learning models designed to identify lung cancer on CXR are highly sensitive.^16^ Other studies have demonstrated strong performance of narrow models designed to detect pneumonia,^17^ pneumothorax,^18^ pneumoconiosis,^19^ cardiomegaly,^20^ pulmonary hypertension^21^ and tuberculosis.^22^ However, narrow models may be problematic in that they draw attention to the presence or absence of the finding they were trained to detect, which may distract the interpreting clinician from other subtle but salient clinical findings.

Comprehensive machine learning models are more clinically useful, removing the need for the application of multiple narrow models and providing valuable information about model performance in images that contain combinations of findings. Some recently developed comprehensive CNN models have demonstrated high performance in identifying a wide range of pathologies on CXR.^23^, ^24^, ^25^ Comprehensive deep learning software can match and exceed the performance of human readers in a non‐clinical environment. One CNN model achieved radiologist‐level performance for 11 of 14 pathologies.^23^ Assessment of a machine learning model capable of detecting 72 findings showed good overall performance compared to radiology residents.^24^ The most comprehensive model validated to date outperformed radiologists in a non‐clinical environment in the detection of 118 findings on chest radiographs and was non‐inferior in a total of 124 findings.^25^ Across all 124 findings, radiologist macro‐averaged AUC was 0.713 and model macro‐averaged AUC was 0.956 when compared to a gold standard of thoracic radiologist panel consensus. It is worth noting that the comprehensiveness of these models lies on a spectrum. A model that detects 14 pathologies simultaneously is likely to be more clinically useful than a narrow model, but is less useful than a model detecting 72 findings, which in turn may have less impact than a model that accurately recognises 124 findings. As machine learning models approach the level of comprehensiveness we expect from highly trained human experts, they are becoming more clinically useful.

After a standalone model performance assessment, the next logical step is to assess whether clinician performance is improved when machine learning software is used to assist interpretation. Narrow models have been shown to improve radiologist diagnostic performance for pneumonia, lung nodules and tuberculosis.^26^, ^27^, ^28^ However, it is in multiple disease detection and comprehensive clinical interpretation where machine learning has the greatest potential to deliver substantial improvements to radiologist performance. Several recent studies have shown significant improvements in radiologist performance when assisted by comprehensive CXR machine learning models. One study assessed a machine learning model used to assist radiology residents, demonstrating improved performance in interpreting chest radiographs obtained in the emergency department setting across a range of findings.^29^ Most recently, the model developed by Seah *et al*.^25^ demonstrated improved radiologist accuracy in over 100 CXR findings comprising a range of acute and non‐acute findings.

## Additional benefits to machine learning in CXR

In addition to assisting in the detection of pathology, machine learning has the potential to improve quantitative assessment, such as volumes and distances. These include estimates of lung nodule size or density, positioning of lines and tubes relative to anatomical landmarks, lung volume or cardiothoracic ratio estimates.^30^ Machine learning systems are well suited to facilitating these tasks, providing accurate estimates quickly without requiring substantial human input. They contribute additional useful clinical information without the cost of workflow disruption. On a similar theme, bone and calcium suppression to improve lung and mediastinal visibility on CXR studies is typically performed by dual‐energy X‐ray machines, requiring specialised hardware.^31^ Deep learning‐based bone suppression systems may offer similar benefits, increasing soft tissue conspicuity and improving the diagnostic accuracy of clinicians,^32^ but solely through software, which is much more accessible, particularly in low‐resource settings.^33^

In addition to improving radiologist accuracy, machine learning models have the capacity to integrate with workflow systems to triage studies, identifying and serving high priority, time‐sensitive findings for faster reporting. Studies suggest that these systems reduce reporting time and alleviate radiologist workloads.^28^, ^34^, ^35^ Triage functionality is likely to become more effective as machine learning solutions become more comprehensive; a model that only looks for a single finding (such as pneumothorax) can up‐triage cases where that pathology is identified, however, in doing so it will necessarily down‐triage cases with other serious or urgent problems (such as free sub‐diaphragmatic gas).

## Limitations

Machine learning systems, however, are susceptible to their own limitations.

The generalisability of trained machine learning algorithms is a salient issue. Models may perform well in one context and poorly in another because of variations in imaging infrastructure, patient population characteristics, disease distributions and overfitting.^36^ This may lead to over‐ or under‐estimation of clinical findings in some population subgroups or in different clinical environments. The data set used to train a model must be carefully selected and should reflect the patient population to which the model will be applied.

Expanding the use of a machine learning model into populations of patients and disease spectrums different to those represented by the training data set is contingent on responsible consideration of the evidence underpinning the model. Broad generalisability needs to be tested in different local populations, and across a variety of different diseases and clinical contexts.^37^, ^38^ Clear and clinically relevant language within machine learning model accuracy validation studies, including describing the patient and disease distributions in the training and test data sets, as well as the potential limitations of model applicability and error rates, is likely to help set appropriate expectations for clinical users. Further research to support the application of machine learning models in wider patient and disease populations is ongoing.

**Fig. 1:**
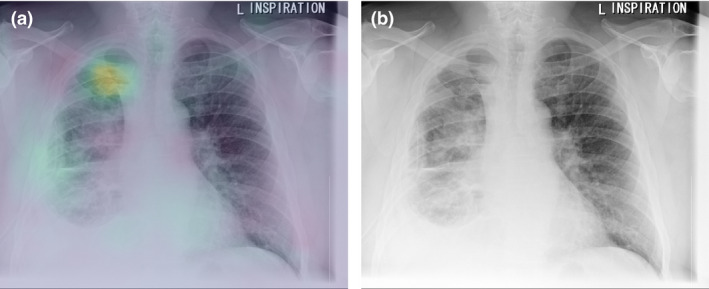
(a) Heatmap investigating an exemplar algorithm^25^ in classifying pneumothorax, demonstrating its focus on the right apical pneumothorax rather than the right‐sided intercostal drain. (b) Original image demonstrating right apical pneumothorax.

Evidence demonstrating model accuracy and clinician performance improvement must be robust as the mechanisms of a trained algorithm’s decision‐making processes are often opaque.^39^ In CXR interpretation, where some findings may be subjective, model opacity may lead to over‐ or under‐confidence in generated results. This may reduce support for system implementation and degrade clinician adoption.^40^ Research on improving algorithm interpretability is generating useful potential solutions.^41^, ^42^ Interrogating black box models to assess the reasons for their conclusions can be useful in minimising internal system bias. One method for assessing the areas in an image given most attention by a machine learning model is to visualise a heat map overlaid onto image pixels. This graphs the attention given to each region of the image over the multiple layers of the model network (Fig. 1). However, heatmaps are often difficult to interpret and may be misleading.^43^

One well‐known consequence of low machine learning model transparency is hidden stratification. Hidden stratification is a phenomenon that can lead to poor model performance on clinically important patient subsets. While a system may demonstrate high performance overall on a broad disease category, it may perform poorly on clinically meaningful subtypes^44^ and lead to failure to detect high‐risk pathologies. The archetypal example is that of detecting pneumothoraces without chest drains. As chest drains are visually obvious and inserted to treat pneumothoraces, deep learning models trained to detect pneumothoraces often rely on the absence or presence of the chest drain to achieve high performance. However, when tested on the clinically relevant subset of pneumothoraces without chest drains, they often perform poorly.^45^

Analysing model performance in clinically relevant subsets of cases is therefore important. This may be difficult if labelling of the training and test data sets is not sufficiently detailed to distinguish between subsets of cases^46^ and if there is inadequate disease variation in the training data set.^44^ Recent evidence suggests that training a machine learning model to both classify the presence of a finding and to provide a localisation (or segmentation) overlay map can reduce the effect of hidden stratification.^45^ High‐quality CXR interpretation systems intended for implementation will need to appropriately address hidden stratification. The development of comprehensive models that record concurrent relevant findings and improved labelling of training data sets will help.

It has been difficult to obtain large data sets with high‐quality, comprehensive labels for model training. While natural language processing techniques are often used to transform free‐text reports into categorical labels, these may be inaccurate and subject to bias,^46^ partly due to variations in language used in reports. The lack of standardisation in medical terminology and approach to reporting are significant challenges that have inhibited the widespread implementation of structured reporting.^47^ RECIST is one example.^48^ Structured reporting has been an aspiration for radiology professional bodies for many years.^49^

## Considerations in machine learning CXR implementation

The rapid evolution of CXR machine learning technology and associated evidence to support its implementation has not yet translated well into widespread adoption. High performing but narrow scope systems produce questionable value for clinical users. This has underpinned pessimistic implementation predictions. However, the development of comprehensive models and evidence demonstrating their real‐world clinical effectiveness in both workflow triage and improved clinician reporting performance appears to be changing this landscape.

As clinical use becomes more widespread, the development of guidelines and professional standards to address acknowledged risks associated with machine learning systems in radiology safeguards patient safety.^40^, ^50^, ^51^ Teams developing, testing and validating machine learning decision support tools should apply these frameworks. Clinicians implementing and using machine learning systems must carefully consider how they were developed and evaluated, including the nature of the training and testing data set populations, generalisability to their own clinical practice and clinical relevance of model outputs.

Regulatory frameworks across global jurisdictions are variable. This may drive delays in achieving clearance for clinical use. Regulatory variations may lead to variations in tool accessibility, which in turn may lead to discrepancies in patient care and outcomes. This is especially relevant for comprehensive models that assess a large number of findings, as thorough testing across all findings is required. Small variations in regulatory requirements may require significant effort to satisfy. There is a need for global harmonisation of regulatory frameworks and the development of guidance and standards in relation to good machine learning practices as well as a common minimum standard of evidence to facilitate innovation and implementation while ensuring patient safety.

In addition to these considerations, other factors have, until now, acted as barriers to the widespread implementation of CXR decision support. These include variable awareness of machine learning models across the radiology community, apprehension regarding cost and implementation complexity, patient privacy protection concerns, and uncertain liability for suboptimal clinical outcomes.^52^ However, as evidence accumulates suggesting performance improvements associated with machine learning‐augmented reporting, the case for the implementation and use of CXR machine learning systems becomes more compelling.

## Looking to the future

Comprehensive image analysis and workflow augmentation comprise the new frontier in applied CXR machine learning practice. It includes systems trained to detect many clinical findings simultaneously, effective calibration to mitigate hidden stratification and generalisation issues, integration into clinical information systems with minimal workflow disruption and identification of time‐sensitive findings for faster report generation. The best of these systems promise to augment radiologist clinical performance and increase efficiency. In future, models that effectively incorporate patient‐specific information as input (e.g. clinical history and previous imaging) will provide more nuanced and tailored output, facilitating the advancement of precision medicine.

The quality of clinical machine learning decision support systems is dependent on the quality of the full product development lifecycle, from initial design to post‐implementation monitoring. Careful data curation and processing are required to ensure that data is broadly representative of clinical populations, to manage label fidelity and to ensure quality model training and validation.^53^ Robust clinical evidence is required to demonstrate reliability, validity, safety and beneficial clinical impact. Usability and interpretability for clinical end users are critical to adoption, and effective post‐implementation performance and safety monitoring is key to quality management and ensuring patient care improvement.

Machine learning is undoubtedly part of the future of radiology, which may require a shift in mindset regarding achievable outcomes, and careful consideration of how to mitigate possible harms. Being a part of the machine learning development process and driving the implementation of high‐quality machine learning systems will be a key responsibility and motivator for radiologists. Part of the role of clinicians is to demand quality machine learning systems and to hold these products to high clinical standards. Radiologists are the guardians of clinical excellence and will play a key role in quality control as powerful and mature machine learning systems begin to filter into clinical practice for the benefit of patients.

## Data Availability

Data sharing not applicable – no new data generated.
